# Improving community mobilization in HIV treatment management: practical suggestions from patients in Brazil

**DOI:** 10.26633/RPSP.2017.119

**Published:** 2017-11-17

**Authors:** Celline Cardoso Almeida-Brasil, Maria das Graças Braga Ceccato, Maria Inês Battistella Nemes, Mark Drew Crosland Guimarães, Francisco de Assis Acurcio

**Affiliations:** 1 Universidade Federal de Minas Gerais, School of Medicine Department of Social and Preventive Medicine, Belo Horizonte Minas Gerais Brazil Universidade Federal de Minas Gerais, School of Medicine, Department of Social and Preventive Medicine, Belo Horizonte, Minas Gerais, Brazil.; 2 Universidade de São Paulo, School of Medicine Department of Preventive Medicine, São Paulo São Paulo Brazil Universidade de São Paulo, School of Medicine, Department of Preventive Medicine, São Paulo, São Paulo, Brazil.

**Keywords:** HIV infections, highly active antiretroviral therapy, medication therapy management, medication adherence, community participation, Brazil, Infecciones por VIH, terapia antirretroviral altamente activa, administración de terapia de medicación, cumplimiento de la medicación, participación comunitaria, Brasil, Infecções por HIV, terapia antirretroviral de alta atividade, conduta do tratamento medicamentoso, adesão à medicação, participação da comunidade, Brasil

## Abstract

**Objectives.:**

*To describe patients’ suggestions on improving the management of antiretroviral therapy (ART) and to identify the roles that key stakeholders should play in taking responsibility for those recommendations*.

**Methods.:**

*This research was embedded within a national cross-sectional study on patient adherence to ART and the associated factors. A subsample of the study patients were asked to offer suggestions on how to improve daily management of ART, and their answers were analyzed using a content analysis approach. The recommendations were then interpreted in terms of who should be responsible for them, and the suggestions were organized into three levels: micro (patient), meso (health care team), and macro (researchers, policymakers, family, friends, and the general public)*.

**Results.:**

*Of the 552 participants from the subsample, 60% were male, their average age was 44.1 years, and 62% were nonadherent in at least at one of three dimensions (missing doses, improper timing, or improper dosing). The categories underlying the micro level were “believing in treatment efficacy,” “being motivated,” “accepting HIV status,” and “sharing experiences with other patients.” At the meso level the suggestion categories were “more information from health care providers” and “humanization of care.” The macro level categories were “social support and actions against stigma,” “research proposals,” and “improvement of health care services*.”

**Conclusions.:**

*Patients are influenced by the health policies, care, and support offered by health care providers, organizations, policymakers, and communities. In turn, these stakeholders develop the policies and deliver their care and support based on the responses and actions of patients. All stakeholders should work together to engage, educate, and support patients in addressing ART management*.

## RESUMEN

**Objetivos:**

Describir las sugerencias de los pacientes sobre cómo mejorar el manejo del tratamiento antirretroviral (TAR) y determinar qué funciones deben desempeñar los interesados directos clave al asumir las responsabilidades con relación a estas recomendaciones.

**Métodos:**

Esta investigación se realizó como parte de un estudio transversal nacional sobre el grado de cumplimiento del tratamiento antirretroviral por parte de los pacientes y los factores asociados. A una submuestra de los pacientes del estudio se le solicitó que ofreciera sus sugerencias sobre cómo mejorar el manejo diario del tratamiento antirretroviral y las respuestas se analizaron mediante un enfoque de análisis de contenidos. Luego se analizaron las recomendaciones en función de quién debería asumir las responsabilidades al respecto y las sugerencias se organizaron en tres niveles: micro (paciente), meso (equipo de atención de salud) y macro (investigadores, responsables de formular políticas, familia, amigos y el público en general).

**Resultados:**

De los 552 participantes de la submuestra, 60% eran hombres, la edad promedio fue de 44,1 años y el 62% no siguieron el tratamiento en al menos una de las tres dimensiones (se saltaron alguna dosis o seguían horarios o dosificaciones inadecuados). A nivel micro, las categorías se referían a “creer en la eficacia de tratamiento”, “estar motivado”, “aceptar el estado con respecto a la infección por el VIH” e “intercambiar experiencias con otros pacientes”. En el nivel meso, las sugerencias entraban en las categorías “recibir más información por parte de los prestadores de atención de salud” y “humanizar la atención”. Las categorías del nivel macro fueron “apoyo social e iniciativas contra el estigma”, “propuestas de investigación” y “mejorar los servicios de atención de salud”.

**Conclusiones:**

Los pacientes reciben la influencia de las políticas en materia de salud, la atención de salud y el apoyo ofrecido por prestadores de atención de salud, organizaciones, comunidades y los responsables de las políticas. A su vez, los interesados directos formulan políticas y prestan su asistencia y apoyo con base en las respuestas y las iniciativas de los pacientes. Todos los interesados directos deben colaborar para involucrar, educar y apoyar a los pacientes en el manejo del tratamiento antirretroviral.

## RESUMO

**Objetivos.:**

Descrever as sugestões dos pacientes para melhorar o manejo da terapia antirretroviral (TAR) e identificar os deveres dos principais interessados diretos ao assumir a responsabilidade por estas recomendações.

**Métodos.:**

Esta pesquisa faz parte de um estudo transversal nacional sobre a adesão dos pacientes à TAR e fatores associados. Foi pedido a uma submostra dos participantes do estudo que fizessem sugestões de como melhorar o manejo diário da TAR. As respostas foram analisadas com um enfoque de análise do conteúdo. As recomendações foram interpretadas segundo a quem cabe a responsabilidade por elas e as sugestões foram organizadas em três níveis: micro (paciente), meso (equipe de saúde) e macro (pesquisadores, responsáveis por políticas, familiares, amigos e o público em geral).

**Resultados.:**

Dos 552 participantes incluído na submostra, 60% eram do sexo masculino, com idade média de 44,1 anos, e 62% não aderiam a, pelo menos, uma das três dimensões (deixar de tomar a dose, tomar o medicamento em horário inadequado ou usar a dose incorreta). As categorias do nível micro foram: ‘’acreditar na eficácia de tratamento, ‘’estar motivado’’, ‘’aceitar ser portador do HIV” e “partilhar experiências com outros pacientes”. Ao nível meso, as categorias de sugestão foram: “mais informações dos profissionais da saúde” e “humanização da atenção”. As categorias do nível macro foram: “apoio social e medidas para combater o estigma’’, ‘’propostas de pesquisa” e “melhoria dos serviços de saúde ”.

**Conclusões.:**

Os pacientes são influenciados por políticas e atenção de saúde e apoio prestado pelos profissionais da saúde, organizações, responsáveis por políticas e comunidades. Por sua vez, os interessados diretos elaboram as políticas e prestam atenção e apoio de acordo com as respostas e ações dos pacientes. Todos os envolvidos devem trabalhar juntos para comprometer, educar e apoiar os pacientes no manejo da TAR.

HIV infection has become a manageable chronic condition since the introduction of potent and safe antiretroviral therapy (ART), which has reduced HIV-associated morbidity and mortality (1). In Brazil since 2013, all HIV-infected individuals are recommended to initiate ART, free of charge, regardless of their CD4 cell count (2). More recently, this recommendation was also put forth by the World Health Organization (WHO) (3). As a result, the number of people living with HIV/AIDS (PLWHA) who are receiving ART has increased, and so has the concern with maintaining adequate levels of adherence over the lifetime course of a chronic disease (3).

Effective management of ART is one of the most important aspects of HIV care. Missing even a few doses can lead to an increase in viral replication, with consequent increased risk of HIV transmission and reduced immunological response. This, in turn, affects the economic burden of the disease and the patient’s quality of life (4). A variety of strategies to improve ART management and adherence have been proposed, including cognitive and behavioral strategies, social support networks, improvement of patient-provider relationships, and simplification of ART regimens (5). Despite these efforts, ensuring adherence to HIV treatment remains challenging in all countries. A meta-analysis of observational studies from 12 different countries showed that only 62% of patients on ART reported optimal adherence (6).

There is a general agreement that community support is associated with better ART outcomes, including optimal adherence (7). Community mobilization is one of the priority areas of the *Treatment 2.0* initiative, which was introduced by the WHO and the United Nations Program on HIV/AIDS (UNAIDS) to maximize the benefits of ART (8). The initiative recommends the involvement and engagement of PLWHA and other key stakeholders in the planning, delivery, and evaluation of HIV treatment and care programs (8). Defined as an individual or group responsible for or affected by health care–related decisions, key stakeholders include patients, providers, policymakers, researchers, and the general public (9). Although the *Treatment 2.0* initiative was launched in 2010, many challenges remain. Community mobilization has been widely studied, but mostly as an intervention for HIV prevention among vulnerable populations (10, 11). In addition, a recent study in Latin America found that compliance with the initiative recommendations on community mobilization averaged just 50% (12).

The Brazilian, Ministry of Health makes continuing efforts to increase social participation in discussion and formulation of HIV/AIDS policies (2). However, it still lacks guidance on implementing WHO recommendations on community mobilization. It is necessary to understand and establish the role of each stakeholder in ART management. In addition, patients’ perspectives can add new insights to complement ART program design and delivery, as well as policy development. However, studies focusing on ART management from the perspective of patients usually identify the problems but without tackling them and proposing practical solutions. The aims of this study were to describe Brazilian patients’ suggestions on how to improve management of HIV treatment and to then identify the roles that key stakeholders should play in taking responsibility for those recommendations.

## METHODS

### Study design and participants

Our study was embedded within a national cross-sectional study entitled Adherence to Antiretroviral Treatment of HIV/AIDS in People under Care at Public Health Centers in Brazil: AVANT. The AVANT study was conducted between December 2009 and December 2011, and its main purpose was to develop an adherence monitoring system (WebAd-Q) (13) and to estimate the prevalence of patient adherence to ART and the associated factors.

The AVANT study included HIV-infected individuals, 18 years old or older, on ART and under care in outpatient facilities at public health care centers. The health care centers were selected following a proportional stratified random sampling from the five geopolitical regions of Brazil (i.e., North, Northeast, Central-West, Southeast, and South). Fifty-five health care centers were included. In each center, the patients were selected through simple random sampling (13). A total of 2 424 participants answered the WebAd-Q, and a subsample of 600 persons (selected randomly and representing 17 health care centers) were invited to participate in our study on how to improve the daily management of ART.

### Procedures

Ethical approval for the AVANT study was obtained from the Research Ethics Committee of the University of São Paulo, School of Medicine. All the participants signed an informed consent form prior to entry into the study.

Data for our study were collected through face-to-face interviews, using a semistructured questionnaire with 58 questions. All interviews were conducted in private rooms and were coded by number for anonymity. The 600 patients from the subsample were asked to offer suggestions on how to improve daily management of ART. Data on sociodemographic and treatment characteristics were also collected. ART nonadherence was accessed through the three questions (on missing doses, taking doses at improper time, or taking improper doses) from the WebAd-Q and was coded into “zero” nonadherence (i.e., answered “no” to all three questions) or nonadherence in one, two, or three dimensions. Each interview lasted around 20 minutes.

### Data analysis

All 600 participants were interviewed, regardless of data saturation, and the total corpus was used for analysis. The answers were recorded during the interviews using written note-taking, with the text returned to the participants for any needed corrections. Field notes were also made during the interviews.

The data were stored and analyzed manually in Excel software, using a content analysis approach (14). Following familiarization with initial data, a coding system of categories was developed based on the open-ended question (i.e., “What do you think can be done to improve ART management?”), study objectives, and the ideas that emerged from the data. Thematic concepts were compared and discussed in meetings between two researchers until a consensus was reached on which categories were most representative of the patients’ responses.

We researchers interpreted the suggestions in terms of who should be responsible for them and then organized that information into a framework of three levels, considering the stakeholders’ interests and scope of influence: micro level (individual), meso level (health care team), and macro level (broader policies and social contexts) (15, 16). This framework has been used to improve the process of shared decision-making (15) and the care for chronic conditions (16), based on the idea that better outcomes are achieved when a health care triad is formed. Since the macro level supports the triad, when its integration is optimal, it creates a positive policy environment for the health care team, which in turn engages patients to become active participants in their care (16). Our findings are illustrated below with representative quotes.

## RESULTS

Of the 600 patients invited to participate, 2 were excluded from the larger study, 28 refused to answer the question on how to improve daily management of ART due to fatigue (given that that was the last question of the long, semistructured questionnaire), and 18 reported not knowing what to answer. All of these 48 patients were excluded from our study, resulting in a response rate of 92% (*N* = 552). Of the 552 participants, 60% were male, 48% had an elementary education or less, and their average age was 44.1 years. The average ART duration for the participants was 7.5 years, and 62% of them were nonadherent in at least at one dimension (Table 1). There were no significant differences with regard to sociodemographic characteristics and adherence between the 552 participants and the 48 nonparticipants. The nonparticipants had a mean age of 43.2 years, and 63% of them were male. In terms of adherence, 63% of the nonparticipants were nonadherent in at least at one dimension.

**TABLE 1.** Descriptive characteristics of people living with HIV participating in the study of suggestions to improve daily management of antiretroviral therapy, Brazil, 2009–2011

Characteristic	Number^a^	%
Age (years old)		
18–40	216	39
41–60	301	55
61–80	35	6
Sex		
Male	332	60
Female	220	40
Highest level of education		
Elementary graduate or less	254	48
Secondary graduate or some secondary	179	34
Post-secondary graduate or some post-secondary	101	18
Duration of antiretroviral treatment (months)		
≤ 12	64	12
13–60	142	25
61–120	203	37
≥ 121	143	26
Nonadherence^b^		
“Zero”	208	38
One dimension	156	28
Two dimensions	128	23
Three dimensions	60	11

a Numbers may not add up to 552 due to missing data.

b The three nonadherence dimensions were: missing doses, improper timing, and improper dosing.

***Source:*** Prepared by the authors from the study data.

The suggestions on how to improve management of ART were coded into nine categories, which were then apportioned among the three levels of stakeholders (Table 2). Some participants (3.3%) said that everything was great and nothing else needed to be done to improve patients’ management of ART, so these answers were not coded.

**TABLE 2.** Suggestions reported by the participants on how to improve management of antiretroviral therapy (ART), Brazil, 2009–2011

Stakeholder level	Category of suggestions	Frequency (no.)
Micro	Believing in treatment efficacy and learning to manage ART	105
	Being motivated	64
	Accepting HIV status	60
	Sharing experiences with other patients	21
Meso	More information from health care providers	47
	Humanization of care	39
Macro	Social support and actions against stigma	162
	Research proposals	141
	Improvement of health care services	77

***Source:*** Prepared by the authors from the study data.

### Micro level

Suggestions that should be addressed by the patients themselves were arranged at the micro level. The categories underlying this level were “believing in treatment efficacy,” “being motivated,” “accepting HIV status,” and “sharing experiences with other patients.”

With regard to the first category, participants reported that consistent behavioral performance, such as adhering to ART as prescribed, creates a habit. Therefore, they seem to understand that compliance provides an advantage, given that it extends one’s life, prevents opportunistic infections, and controls the virus. A 51-year-old man said, “We need to strictly follow the physician’s instructions. All the patients should be aware that treatment increases our quality of life.”

Being motivated was mentioned as a result of being optimistic and religious, having a sense of self-worth and a strong will to live, finding reasons to live, and keeping the mind busy. A 45-year-old woman said, “Enjoy your life, go to parties, play sports, keep yourself in a good mood.” And a 53-year-old woman stated, “You can’t isolate yourself and feel useless. Have faith in God and never lose hope.”

Accepting HIV status was noted by some participants as an important strategy to manage ART and achieve a “normal” life. By accepting the diagnosis, problems such as denial and anger would no longer be barriers to ART adherence. A 71-year-old man said, “Do not fear, do not hide, face it, and live a normal life. It’s a disease like any other.” However, even after accepting the diagnosis, patients still may be victims of stigma, thus the suggestion of some patients was to “keep to yourself that you are HIV-positive” and “don’t walk around talking about it with anyone.”

Joining support groups to share information on facilitators and barriers of ART was mentioned as a way to learn from the experience of others. A 45-year-old man said, “Patients should motivate themselves with successful stories of others like me, for example. I’ve had no problems on the job or with discrimination.” Participants also demonstrated a desire to participate in events with other patients outside the health care facilities, by saying that “people with HIV should not hide, they should meet in activities and events out of the health care center.”

### Meso level

Suggestions related to health care professionals (e.g., physicians, nurses, and pharmacists) were included in the meso level and arranged between the categories “more information from health care providers” and “humanization of care.”

Participants mentioned problems related to communication with the provider. For example, they said they had not received sufficient information, even the basics, in order to understand their disease or the treatment. A 27-year-old man stated, “We need more clarification on what the medicines do to us. The physician needs to educate us about the disease and the side effects. Also, we need to know what happens if we stop taking the pills.”

Participants also demanded the humanization of care. This issue was expressed in terms of having a poor personal connection with the provider, feeling excluded from the decision-making process, and feeling stigmatized by health care providers. Participants insisted on “sufficient time with the provider during appointments,” “more interaction and personalized treatment,” “less prejudice from professionals,” “more adherence incentives from doctors,” “participation in decisions,” and “not being seen as a number.” Some participants thought that increased payments for doctors would be a way to improve the patient-provider relationship, as the following quote from a 37-year-old man indicated: “Without the physician, we cannot get there. They [the providers] should receive more money, so they could treat us better.”

### Macro level

The macro level includes suggestions for researchers, policymakers, and people from the patients’ living and working environments. The codes were organized into the following categories: “social support and actions against stigma,” “research proposals,” and “improvement of health care services.”

Others’ stereotypical thinking about HIV may represent a barrier to coping with ART. As a 33-year-old woman said, “There are many people who discriminate against people with HIV. If there weren’t people like that, it would be easier to succeed.” Many participants indicated that the problem of stigma should be addressed through provision of appropriate education to patients and communities and that stigma-reducing interventions should reach as many people as possible, as a 37-year-old woman pointed out: “It could be shown on TV that ‘normal’ people should not fear being around people living with HIV.” When PLWHA internalize the stereotypes and discrimination toward them, they may isolate themselves and receive limited social support. Therefore, participants suggested that their family and friends should approach them and offer support to make them feel accepted, respected, cared for, and motivated. A 60-year-old man said, “We need more understanding from people who are close to us. We need these people to encourage us, to boost our morale. Talking solves everything.”

A quarter of the participants demonstrated that they have their own perspective on what should be investigated in HIV research. Their wishes were linked either to their perceptions that antiretroviral drugs are easily identified by other people (thus contributing to the participants’ fear of stigmatization) or to the fact that they are apprehensive that ART will stop working or will lead to negative effects in the future. A 42-year-old woman suggested there should be new drugs “that are easier to take, like some liquid or injectable, or even fewer pills, without side effects.” Participants also expressed a desire for “urgently finding a cure.” They stressed the importance of research on ART management, but they also said that disseminating research results did not necessarily increase patients’ understanding of ART. However, some participants did express an interest in taking part in academic conferences.

Participants pointed out some characteristics of the outpatient facility that could be improved. They feared that other patients at the health care center could learn about their HIV-positive status and suggested that “patient identification should be done by numbers, never by names.” Other complaints included long wait times to get test results and the absence of other medical specialists, such as psychiatrists and cardiologists. The exchange of information between medical specialties was commonly perceived as insufficient, as expressed by a 37-year-old man: “There is a lack of coordination. All specialties should be integrated into the same facility. As for me, I have vascular disease, but I can’t get it treated here.” Participants also suggested that the appointment system be revised, allowing them to reach a physician whenever they need one, or at least to have a shorter interval between appointments.

## DISCUSSION

This study gave us a deeper understanding of HIV patients’ perceptions of what should be done to improve the management of ART and of the key stakeholders’ role in the delivery and management of HIV care and treatment. Almost two-thirds of the participants were nonadherent in at least at one of the three dimensions (i.e., missing doses, improper timing, or improper dosing). This demonstrates the importance of our results for implementing community mobilization, where key stakeholders would incorporate the patients’ suggestions in their area of influence and propose actions to improve ART management.

**FIGURE 1.**
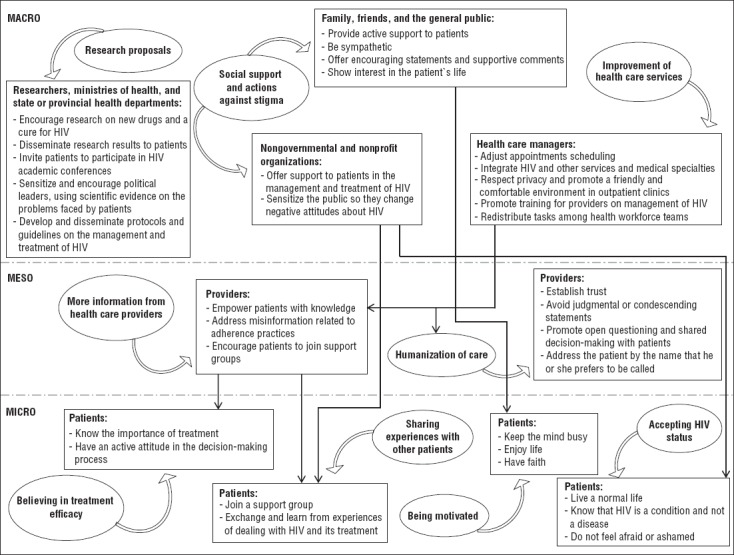
Dynamic framework of patients’ suggestions for key populations to improve antiretroviral therapy management, Brazil, 2009–2011

***Source:*** Prepared by the authors from the study data.

We interpreted the patients’ suggestions in terms of who should be responsible for them, and we then organized those recommendations into the three levels: micro, meso, and macro. Each of these levels interacts with and influences the other two. Patients are influenced by the health policies, care, and support offered by health care providers, organizations, policymakers, and communities. In turn, these stakeholders develop the policies and deliver their care and support based on the responses and actions of patients (16). A framework of the suggestions and their interpretation is given in Figure 1 and is discussed below.

The participants suggested to other patients that they should believe in treatment efficacy and adhere to ART. Curioso et al. (17) observed that faith in treatment was related to increased self-motivation for adherence and the integration of treatment into one’s life. Patients should be empowered with more knowledge about the treatment, especially its importance and the side effects it may have. This requires interventions from health care providers to help patients initiate new behaviors, such as self-management techniques and lifestyle changes (16). Patients know that providers play a key role in treatment management, but sometimes they may feel insecure and lose trust when the providers fail to provide all information that the patients need, thus negatively influencing their adherence (18, 19). Nurses and pharmacists should provide information to patients, in consonance with the task-shifting tool devised by WHO (20). The approach involves the rational redistribution of tasks among health workforce teams in order to address the burden that HIV infection imposes on the health care system (20).

The term “humanization of care” includes the recognition of the value of patients, workers, and managers of the health care system (21). However, the participants in our study demanded the humanization of care solely in terms of the circumstances of the patient-provider relationship. Although relationships are built through effective communication and interaction, in the Brazilian health care centers, patients are not consistently scheduled with a specific provider. This prevents the adequate contact and bonding with a provider that could help build a personal relationship. Tabler et al. (22) observed that patients think that short appointments indicate that the provider is more interested in making money than in taking care of them. This is a reasonable explanation for why participants in our study thought that better monetary rewards for providers would improve the quality of their care. In order to achieve personalized care and build mutual trust, providers should respect patients’ preferences and preserve their dignity. Providers should also be trained to communicate effectively and to promote information exchange and open questioning (18). In addition, providers need to guarantee a shared decision-making process with patients, since the National Humanization Policy (NHP) in Brazil recommends that every key population, including patients, play a central role in health events (21).

Another principle of the NHP is the inseparability of care and policies in regard to the organization and infrastructure of health care services (21). When asking for improvement in the health care services, our study’s participants focused on the functional aspects of the health service quality, i.e., the manner in which services are delivered to them. The fear of disclosure due to lack of privacy in outpatient clinics that was mentioned by participants was also noted by patients in Tanzania as a factor that hampers adherence to ART (23). Health care managers should tailor services to the context and culture of patients, respecting privacy and promoting a friendly and comfortable environment. As one study participant suggested, patients waiting at health care centers could be identified by their medical record number instead of their name. Participants’ demands for more frequent appointments or for more providers were also observed in a study about patients’ perceptions of quality in primary health care (24). However, the lack of public financial resources to improve the physical structure and material services limits the strategies to respond to the patients’ wishes. Nonetheless, the task-shifting tool mentioned above could be a partial solution to physician shortages. Another strategy, the integration of health care services, may lead to improved health, less waste, decreased inefficiency, and more satisfaction among patients (16). If it is not possible to integrate some specialties into the same center, the system could at least be integrated so the physicians could access patient records from other specialists. The patients’ complaints about the service quality can offer managers guidance for improving their organizations.

Participants suggested to other patients that they should create patient groups and share their experiences in dealing with HIV and ART. Peer support among patients can improve adherence to long-term therapies and reduce the load on health care providers (25). Those providers should encourage patients to join support groups, especially ones managed by community leaders in nongovernmental organizations (NGOs). A study conducted in Ecuador found that patients who contacted an HIV/AIDS NGO were more likely to better deal with their HIV status (26). Accepting HIV status is related to improved adherence to ART (27), but it is also negatively influenced by the way patients internalize the HIV-related stereotypes and discrimination they face (28). Another important role for community leaders and NGOs is encouraging the public to change their negative attitudes regarding HIV infection. Strategies to do that could include distributing written information and disseminating PLWHA personal-experience chronicles in the media (29), which could influence institutions and groups at the community level (30).

Although studies often recommend that PLWHA should be encouraged to join support groups (26, 28), our study found that patients prefer the contrary approach, expecting an active attitude from family, friends, and the public in general. Consistent with this finding, Teoh et al. (31) found that active social support provided by friends (that is, friends taking the initiative in giving supportive comments and gestures) produces a higher level of positive affect than does passive support (that is, friends showing no interest and ignoring the patient). Family, friends, and the general public should provide social support by showing genuine interest in the patient, maintaining eye contact and adopting an open body posture, and offering encouraging statements and supportive comments (18). Social support reduces anxiety and depression symptoms among PLWHA and is associated with a number of positive outcomes such as hope and optimism (32). Hope and optimism were in turn mentioned by the participants as motivators for better ART management. The will to live, which is motivated by such factors as family, partners, job, and religion, requires that patients take care of their own health, thus improving adherence to ART (33).

Principal investigators often decide what should be explored in a specific field of research, and this may not match the patients’ wishes. A recent study that asked Parkinson’s disease patients about their priorities for research found their concerns focused on the cause and cure of the illness, medication, and good care (34). Similarly, our study found that patients asked mainly for research on new drugs and on curing HIV. The concern regarding the pill burden has already been addressed, and nowadays most people who start ART in many countries, including Brazil, take the single-tablet regimen (35). However, those who initiate their treatment with a regimen other than a first-line one and patients who change their initial regimen may still face problems with pill burden or critical drug adverse effects. One of the goals of the *Treatment 2.0* initiative is to promote the development of effective one-pill, once-daily antiretroviral regimens with minimal toxicities and high barriers to drug resistance (8). Several such new drugs are in the advanced stages of development (35). Investigating patients’ wishes is linked to recognizing their active role in decision-making. Along these lines, researchers should disseminate research results to patients (such as through informational brochures or media videos in lay language) and encourage patients to participate in academic conferences on HIV.

The macro level is where strategies and policies for health care development are traced out. Researchers and decisionmakers should educate political leaders by providing them scientific evidence on the problems that patients face and on proposed strategies and models for managing those difficulties. For their part, political leaders should support epidemiological studies on HIV treatment as well as the development of new antiretroviral drugs. In addition, ministries of health, along with their countries’ state or provincial health departments, should promote the development and dissemination of treatment protocols for the education and training of health care providers in the diagnosis and management of HIV, based on recent scientific evidence.

### Study limitations

There were some limitations in this study. As the qualitative study was part of a bigger quantitative project, some criteria usually used in qualitative research were not applied in this study. For example, we did not use audio or video recording in the data collection, and we did not contact the participants for feedback on our findings. Since all the suggestions were based only on patients’ perceptions, other important concerns about health care services, such as allocation of resources and access to medication, were not covered in our study. Future studies should include focus groups with patients, health care providers, community members, and policymakers in order to collaboratively identify, from different perspectives, what could be done to help patients cope with ART.

### Study strengths

Despite the limitations mentioned above, some study strengths should be cited. Our work represents an attempt to convey patients’ suggestions about ART management to key populations. This study further illustrates topics for workshop discussions among crucial stakeholders. In addition, our findings could be used as a base for projects to be developed by civil society organizations, with an emphasis on treatment adherence and community mobilization against stigma.

### Conclusions

We found that patients expect to be listened to by providers, researchers, policymakers, and community members. Most importantly, our results indicate that patients should play an active role in decision-making; health care providers should deliver personalized care and empower patients with knowledge; health care managers should promote training and continuing education for providers, as well as redistribute tasks among health workforce teams; family, friends, and the general public should provide active social support for patients; nongovernmental organizations should make efforts to sensitize the public and thus reduce stigma; and researchers should disseminate their findings to political leaders and patients. All stakeholders should work together to successfully function in relation to each other and engage, educate, and support patients in addressing ART management.

#### Acknowledgments.

The authors thank all the study participants for having patience and providing valuable information.

#### Funding.

This work was funded by the Department of STD/AIDS and Viral Hepatitis of the Brazilian Ministry of Health.

#### Disclaimer.

Authors hold sole responsibility for the views expressed in the manuscript, which may not necessarily reflect the opinion or policy of the *RPSP/PAJPH* or PAHO.

## References

[1] 1. Cohen MS, Chen YQ, McCauley M, Gamble T, Hosseinipour MC, Kumarasamy N, et al. Prevention of HIV-1 infection with early antiretroviral therapy. N Engl J Med. 2011;365(6):493–505. 10.1056/NEJMoa1105243 PMC3200068 21767103

[2] 2. Brasil Ministério da Saúde. The Brazilian response to HIV and AIDS. Brasília: Ministério da Saúde; 2015. Available from: http://www.unaids.org/sites/default/files/country/documents/BRA_narrative_report_2015.pdf Accessed 15 March 2016.

[3] 3. World Health Organization. Guideline on when to start antiretroviral therapy and on pre-exposure prophylaxis for HIV. Geneva: WHO; 2015. Available from: http://www.who.int/hiv/pub/guidelines/earlyrelease-arv/en/ Accessed 15 April 2016. 26598776

[4] 4. Vandewalle B, Llibre JM, Parienti JJ, Ustianowski A, Camacho R, Smith C, et al. EPICE-HIV: an epidemiologic cost-effectiveness model for HIV treatment. PLoS One. 2016;11(2):e0149007 10.1371/journal.pone.0149007 PMC4752501 26870960

[5] 5. Enriquez M, McKinsey DS. Strategies to improve HIV treatment adherence in developed countries: clinical management at the individual level. HIV AIDS (Auckl). 2011;3:45–51 10.2147/HIV.S8993 PMC3218706 22096406

[6] 6. Ortego C, Huedo-Medina TB, Llorca J, Sevilla L, Santos P, Rodríguez E, et al. Adherence to highly active antiretroviral therapy (HAART): a meta-analysis. AIDS Behav. 2011;15(7):1381–96. 10.1007/s10461-011-9942-x 21468660

[7] 7. Zachariah R, Teck R, Buhendwa L, Fitzerland M, Labana S, Chinji C, et al. Community support is associated with better antiretroviral treatment outcomes in a resource-limited rural district in Malawi. Trans R Soc Trop Med Hyg. 2007;101(1):79–84. 10.1016/j.trstmh.2006.05.010 16962622

[8] 8. World Health Organization. The Treatment 2.0 framework for action: catalyzing the next phase of treatment, care, and support. Geneva: WHO; 2011. Available from: http://www.who.int/hiv/pub/arv/treatment/en/ Accessed 29 February 2016.

[9] 9. Concannon TW, Meissner P, Grunbaum JA, McElwee N, Guise JM, Santa J, et al. A new taxonomy for stakeholder engagement in patient-centered outcomes research. J Gen Intern Med. 2012;27: 985–91. 10.1007/s11606-012-2037-1 PMC3403141 22528615

[10] 10. Kerrigan D, Kennedy CE, Morgan-Thomas R, Reza-Paul S, Mwangi P, Win KT, et al. A community empowerment approach to the HIV response among sex workers: effectiveness, challenges, and considerations for implementation and scale-up. Lancet. 2015;385(9963):172–85. 10.1016/S0140-6736(14)60973-9 PMC7394498 25059938

[11] 11. Sadhu S, Manukonda AR, Yeruva AR, Patel SK, Saggurti N. Role of a community-to-community learning strategy in the institutionalization of community mobilization among female sex workers in India. PLoS One. 2014;9(3):e90592. 10.1371/journal.pone.0090592 PMC3946527 24608680

[12] 12. Perez F, Gomez B, Ravasi G, Ghidinelli M. Progress and challenges in implementing HIV care and treatment policies in Latin America following the Treatment 2.0 initiative. BMC Public Health. 2015;15:1260. 10.1186/s12889-015-2565-9 PMC4684910 26686850

[13] 13. Vale FC. Avaliação do questionário WebAd-Q como ferramenta de monitoramento da adesão ao tratamento antirretroviral nos serviços do SUS [dissertation]. São Paulo: Universidade de São Paulo; 2014. Available from: http://www.teses.usp.br/teses/disponiveis/5/5137/tde-09022015–094334/pt-br.php Accessed 1 February 2016.

[14] 14. Bardin L. Análise de conteúdo. 4th ed. Lisboa: Edições 70; 2008.

[15] 15. Légare F, Stacey D, Pouliot S, Gauvin FP, Desroches S, Kryworuchko J, et al. Interprofessionalism and shared decision-making in primary care: a stepwise approach towards a new model. J Interprof Care. 2011;25(1):18–25. 10.3109/13561820.2010.490502 PMC3018136 20795835

[16] 16. World Health Organization. Innovative care for chronic conditions: building blocks for action: global report. Geneva: WHO; 2002. Available from: http://www.who.int/chp/knowledge/publications/icccglobalreport.pdf Accessed 12 April 2016.

[17] 17. Curioso WH, Kepka D, Cabello R, Segura P, Kurth AE. Understanding the facilitators and barriers of antiretroviral adherence in Peru: a qualitative study. BMC Public Health. 2010;10:13. 10.1186/1471-2458-10-13 PMC2820472 20070889

[18] 18. Frampton S, Guastello S, Brady C, Hale M, Horowitz S, Smith SB, et al. Patient-centered care improvement guide. Derby, Connecticut: Planetree, Inc. and Picker Institute; 2008.

[19] 19. Beach MC, Keruly J, Moore RD. Is the quality of the patient-provider relationship associated with better adherence and health outcomes for patients with HIV? J Gen Intern Med. 2006;21(6):661–5. 10.1111/j.1525-1497.2006.00399.x PMC1924639 16808754

[20] 20. World Health Organization. Task shifting: rational redistribution of tasks among health workforce teams: global recommendations and guidelines. Geneva: WHO; 2008. Available from: http://www.who.int/healthsystems/TTR-TaskShifting.pdf?ua=1 Accessed 10 April 2016.

[21] 21. Brasil Ministério da Saúde. HumanizaSUS: documento base para gestores e trabalhadores do SUS. Brasília: Ministério da Saúde; 2010. Available from: http://bvsms.saude.gov.br/bvs/publicacoes/humanizasus_documento_gestores_trabalhadores_sus.pdf Accessed 12 April 2016.

[22] 22. Tabler J, Scammon DL, Kim J, Farrell T, Tomoaia-Cotisel A, Magill MK. Patient care experiences and perceptions of the patient-provider relationship: a mixed method study. Patient Exp J. 2014;1(1): 75–87.

[23] 23. Lyimo RA, de Bruin M, van den Boogaard J, Hospers HJ, van der Ven A, Mushi D. Determinants of antiretroviral therapy adherence in northern Tanzania: a comprehensive picture from the patient perspective. BMC Public Health. 2012; 12:716. 10.1186/1471-2458-12-716 PMC3488585 22935331

[24] 24. Papp R, Borbas I, Dobos E, Bredehorst M, Jaruseviciene L, Vehko T, et al. Perceptions of quality in primary health care: perspectives of patients and professionals based on focus group discussions. BMC Family Practice. 2014;15(128):1–13. 10.1186/1471-2296-15-128 PMC4083126 24974196

[25] 25. World Health Organization. Adherence to long-term therapies: evidence for action. Geneva: WHO; 2003. Available from: http://apps.who.int/iris/bitstream/10665/42682/1/9241545992.pdf Accessed 15 March 2016

[26] 26. Bernier A, Acosta ME, Castro DR, Bonifaz C, Jaramillo S, Henry E, et al. Factores asociados a establecer contacto con asociaciones de lucha contra el VIH/sida en Ecuador: resultados de un estudio comunitario. Rev Panam Salud Publica. 2015;38(3):209–16. 26757999

[27] 27. Katz IT, Ryu AE, Onuegbu AG, Psaros C, Weiser SD, Bangsberg DR, et al. Impact of HIV-related stigma on treatment adherence: systematic review and meta-synthesis. J Int AIDS Soc. 2013;16(3 Suppl 2): 18640. 10.7448/IAS.16.3.18640 PMC3833107 24242258

[28] 28. Li J, Mo PKH, Wu AMS, Lau JTF. Roles of self-stigma, social support, and positive and negative affects as determinants of depressive symptoms among HIV infected men who have sex with men in China. AIDS Behav. 2016;(Epub ahead of print):1–13. 10.1007/s10461-016-1321-1 PMC4992470 26896120

[29] 29. Sengupta S, Banks B, Jonas D, Miles MS, Smith GC. HIV interventions to reduce HIV/AIDS stigma: a systematic review. AIDS Behav. 2011;15(6):1075–87. 10.1007/s10461-010-9847-0 PMC3128169 21088989

[30] 30. Atkin CK, Rice RE. Advances in public communication campaigns. In: Scharrer E, ed. The international encyclopedia of media studies: vol. 5: Media effects/media psychology. London: Wiley-Blackwell; 2013: 526–51.

[31] 31. Teoh AN, Chia MSC, Mohanraj V. The comparison between active and passive types of social support: the emotional responses. J Appl Biobehav Res. 2009; 14(2):90–102.

[32] 32. Liu L, Pang R, Sun W, Wu M, Qu P, Lu C, et al. Functional social support, psychological capital, and depressive and anxiety symptoms among people living with HIV/AIDS employed full-time. BMC Psychiatry. 2013;13:324. 10.1186/1471-244X-13-324 PMC4219509 24289721

[33] 33. Wasti SP, Simkhada P, Randall J, Freeman JV, van Teijlingen E. Barriers to and facilitators of antiretroviral therapy adherence in Nepal: a qualitative study. J Health Popul Nutr. 2012;30(4):410–9. 10.3329/jhpn.v30i4.13294 PMC3763612 23304907

[34] 34. Schipper K, Dauwerse L, Hendrikx A, Leedekerkern JW, Abma TA. Living with Parkinson’s disease: priorities for research suggested by patients. Parkinsonism Relat Disord. 2014;20(8):862–6. 10.1016/j.parkreldis.2014.04.025 24874526

[35] 35. Cihlar T, Fordyce M. Current status and prospects of HIV treatment. Curr Opin Virol. 2016 March 26;18:50–6. 10.1016/j.coviro.2016.03.004 27023283

